# Strategies to increase demand for maternal health services in resource-limited settings: challenges to be addressed

**DOI:** 10.1186/s12889-015-2222-3

**Published:** 2015-09-08

**Authors:** Khalifa Elmusharaf, Elaine Byrne, Diarmuid O’Donovan

**Affiliations:** Reproductive & Child Health Research Unit (RCRU), University of Medical Sciences & Technology, Khartoum, Sudan; Royal College of Surgeons in Ireland, Manama, Bahrain; National University of Ireland, Galway, Ireland; Royal College of Surgeons in Ireland, Dublin, Ireland

## Abstract

**Background:**

Universal health access will not be achieved unless women are cared for in their own communities and are empowered to take decisions about their own health in a supportive environment. This will only be achieved by community-based demand side interventions for maternal health access. In this review article, we highlight three common strategies to increase demand-side barriers to maternal healthcare access and identify the main challenges that still need to be addressed for these strategies to be effective.

**Discussion:**

Common demand side strategies can be grouped into three categories:(i) Financial incentives/subsidies; (ii) Enhancing patient transfer, and; (iii) Community involvement. The main challenges in assessing the effectiveness or efficacy of these interventions or strategies are the lack of quality evidence on their outcome and impact and interventions not integrated into existing health or community systems. However, what is highlighted in this review and overlooked in most of the published literature on this topic is the lack of knowledge about the context in which these strategies are to be implemented.

**Summary:**

We suggest three challenges that need to be addressed to create a supportive environment in which these demand-side strategies can effectively improve access to maternal health services. These include: addressing decision-making norms, engaging in intergenerational dialogue, and designing contextually appropriate communication strategies.

## Background

Maternal death is defined as “the death of a woman while pregnant or within 42 days of termination of pregnancy, irrespective of the duration and site of the pregnancy, from any cause related to or aggravated by the pregnancy or its management but not from accidental or incidental causes” [1]. Maternal death is a good indicator of the utilisation of health services. It is the ‘tip of the iceberg’ that reveals the magnitude of pregnancy-related conditions, near-miss events, other potentially devastating consequences after birth, and the long-term psychological, social, and economic consequences [2].

There has been marked progress and positive changes in maternal health in some low-income countries [3, 4], however, many campaigns have failed to improve maternal health in the last three decades [5]. This is mainly because of neglected health systems [6]. The Safe Motherhood Initiative, launched in Kenya in 1987 by international agencies [7], is an example of a failure to generate a broad-based improvement in this area of public health because of the inability to translate recommendations into local practice [5, 8].

Lack of knowledge of the importance of seeking medical attention during pregnancy and labour is commonly believed to negatively influence health behaviour and decision making processes. The choice of seeking healthcare is embedded and intertwined with cultural and social practices especially for women in remote rural villages [9, 10]. Women’s education, employment, and affordability are the most commonly identified factors affecting antenatal care uptake [11].

A meta-synthesis of qualitative studies [12] identified important key issues of why women do not use antenatal services in low- and middle-income countries. These include community belief systems that consider pregnancy as socially risky and physiologically healthy that limits the initial access to maternal healthcare. Uptake is also impacted by financial constraints and other physical limitations surrounding patient transfer in conditions of extreme poverty.

The majority of maternal deaths occur at homes in rural areas, among poorer communities and during the peripartum period - the last three months of the pregnancy to the first week after the end of the pregnancy [13]. A peak in maternal mortality occurs during the intrapartum period around childbirth and the first day post-partum [14]. Hence Filippi et al., in the editorial of *The Lancet* series on maternal survival [2], called for a clear strategic vision that prioritises the intrapartum period in order to reduce maternal mortality.

Sufficient data is available on maternal health to inform global action, yet the poorest and most fragile countries have the poorest data to monitor and measure maternal health [13]. Designing a health system that addresses the local situation requires knowledge of the context. The lack of publically available data and limited published literature limit contextually based interventions to improve maternal health in low-income countries.

The main reasons for maternal deaths within the health system are the lack of skilled birth attendants, remoteness, delay in referral for emergency obstetric care [14], delay or poor implementation of interventions at the facility level, and vertical delivery of care in which single elements of care are implemented without connection with the comprehensive care [15].

Maternal health services are dependent on the complex interdependent functioning of the entire health system [16]. The links between inputs, process and outcomes are subject to multiple influences and confounding factors, and each country’s context determine many factors that influence the outcomes of maternal health and the performance of the service [17]. The intermittent nature of demand, the difficulty in accessing quality maternal health services, and the wide range of powerful stakeholders with different priorities and agendas make the health system extremely complex [18]. In addition, international donors may influence the conditions of a country’s health programmes to satisfy their own agenda [6].

Many strategies have been suggested to reduce maternal mortality, including contraception, antenatal care, referral systems that include basic and comprehensive emergency obstetric care, and postnatal care. A recent review of the evidence shows the significant and successful role of family planning as a preventative strategy in reducing maternal mortality [19]. Antenatal care that includes provision of Misoprostol for prevention of postpartum haemorrhage at home births has been proven to be one of the most cost effective interventions to reduce maternal deaths [20].

However, it is now evident that high coverage of essential interventions in healthcare facilities does not necessarily reduce maternal mortality [15] largely due to services not being utilised. Universal health access will not be achieved unless women are cared for in their own communities. Additionally women need their capacity and capabilities strengthened so that they can take ownership of the decisions about their care at the right time and without having to rely or be expected to rely on others to make these decisions for them. Thus a strong focus in attaining universal maternal healthcare access is to overcome demand side barriers.

**Fig. 1:**
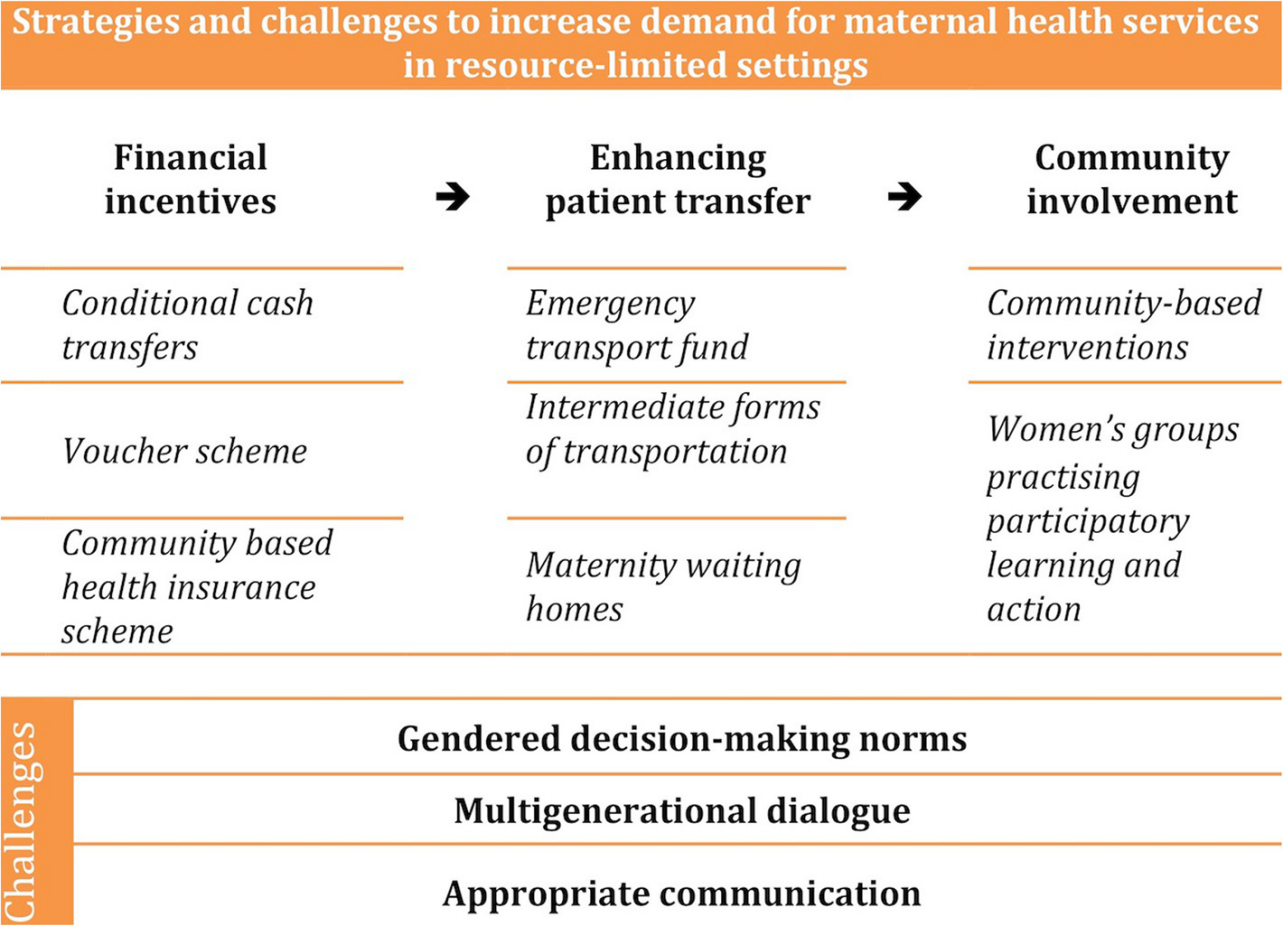
Conceptual framework for strategies to overcome demand-side barriers to maternal healthcare access and their challenges

The aim of this review article is to identify the existing common strategies that have been implemented to overcome demand-side barriers to maternal healthcare access, as well as debating their limitations and challenges. The finding of this review will help to inform the global conversations around universal health coverage and universal health access and to help develop appropriate and sustainable strategies at community level to enhance demand for maternal care. Based on the articles reviewed we can group the main demand-side approaches to improving maternal health service access into: (i) Financial incentives/subsidies; (ii) Enhancing patient transfer, and; (iii) Community involvement (Fig. 1). The main challenges in assessing the effectiveness or efficacy of these interventions or strategies is the lack of quality evidence on their outcome and impact [21–23], as well as the lack of a system-wide interventions or interventions not integrated into existing systems for likely sustainability [24]. However, what is also noted, though not highlighted as much as the above, is the lack of knowledge about the context in which these strategies are to be implemented

## Methods

The initial review was focused on identifying the most common demand side strategies in resource poor settings to improve maternal health services in the published literature. Medline PubMed was initially searched using a combination of the terms: maternal health service; demand or demand-side; low-income/low and middle income/developing country; interventions or strategy. A review of Google Scholar also identified additional articles. In total 26 articles were obtained from an initial 307 articles in PubMed (6 systematic reviews and 20 articles on specific interventions and strategies). An additional 17 articles (2 reviews) were added from Google Scholar. Three Cochrane reviews were also included.

Endnote was used to generate reports listing basic bibliographic data such as title, authors and journal name. Titles were reviewed and screened on the basis of their relevance to the study. After the initial title screen, abstracts were screened. Inclusion criteria were: described or reviewed specific strategies or interventions, as opposed to opinions or proposed strategies; focused on demand side strategies or interventions, and: were specific to maternal health service utilisation. Articles were to be available in English and a time limit of the last 20 years was adhered to. No geographic limitations were used. In total 44 articles were reviewed. However, the purpose of the review was not to be exhaustive in terms of all possible interventions or strategies, but to become familiar with the most common ones described in the published literature.

These documents were supplemented by additional sources identified through ongoing research scanning, reference lists of articles, searches to address gaps in the initial searches and expert recommendations. Web-based resources were searched to supplement the journal articles. The following websites were searched: WHO, World Bank, DFID, USAID, and relief web.

## Discussion

### Strategies to overcome demand-side barriers

Three main categories of demand side intervention can be described: Financial incentives/direct subsidies (by far most published on this); enhancing patient transfer, and; community involvement.

### Financial incentives

This was by far the most common form of demand side intervention addressed in the published literature. The main approaches to improving demand for maternal and reproductive health are: conditional cash transfers; voucher schemes, and community-based and social health insurance. There was some discussion on direct subsidies [23] and the reduction of user fees [25].

#### Conditional cash transfers

Conditional cash transfer is a social protection innovation that provides cash to poor households conditional on meeting health service requirements such as attending perinatal care, growth monitoring, and vaccinations for children or educational conditions such as school enrolment and good attendance [26]. Most of the large-scale conditional cash transfer programmes have been implemented in Latin America. In Mexico [27, 28], Nicaragua [29], and Colombia [30] the focus has been on child health and education, while in Brazil the focus included maternal health as well as child health [31, 32].There is now compelling evidence that conditional cash transfers in general increase income and overall household consumption and nutrition [33]. Furthermore, it promotes the accumulation of human capital among poor households [34] and increases access to healthcare for hard-to-reach groups [35]. Evidence about conditional cash transfer programmes strongly illustrates their effectiveness in improving access to preventative services and, sometimes, in improving health status and maternal health [36, 37]. Nonetheless, it is still not clear whether the various pathways through which the conditional cash transfers work are caused by the structure of this scheme or through behavioural changes [38]. On the other hand, the success of conditional cash transfer programmes in Latin America is not necessarily transferable to other parts of the world and their replicability in poor settings is still uncertain [38]. For example, the conditional incentive programme in Nepal for all pregnant women that encourages institutional delivery faced severe constraints in implementation at district level. These included bureaucratic delays, lack of planning and weak and inadequate health services [39].

#### Voucher scheme

A voucher system has been introduced as a form of demand-side funding in many settings to provide access to pre-defined services and to improve targeting of hard-to-reach populations. These redeemable coupons for a defined service package place the power of purchasing care in the hands of patients [23]. Vouchers have been used for maternal healthcare in Cambodia to improve access, quality and inequities of selected reproductive health services [40]. In India a voucher scheme, which was implemented to increase institutional delivery for emergency obstetric care for the poor, succeeded in providing substantial benefits to poor people [41]. A recent quasi-experimental trial was conducted in Eastern Uganda to study the effect of the voucher scheme on improving institutional delivery and enhancing maternal follow-up. Women in the intervention group were given booklets containing transport vouchers and service vouchers to facilitate access to free transport and free antenatal care, delivery care and postnatal care. Early results show a rapid increase in the utilisation of maternal care [42, 43]. However, while there is growing evidence to support demand-side financing, simply providing vouchers does not guarantee utilisation of services. Issues such as the wider cultural context, inequality, transportation system limitations, cost effectiveness, health system strengthening and sustainability all need to be considered and integrated [23, 43].

#### Community based health insurance scheme

A community-based health insurance (CBHI) scheme is a voluntary form of health insurance that is organised at community level with the principles of risk-pooling and regular payments of a small premium [44]. It aims to prevent catastrophic health expenditure, particularly among the underserved and the poorest of the poor. Community members are involved in the management of the insurance and the selection of the health services it covers [45]. For example, in Senegal, there were 40 functioning CBHI schemes in 2004 in the Thiès region (second largest city in Senegal). The membership enrolment requires the entire nuclear families to join the scheme with a monthly premium of $0.20 to $0.40 per individual per month. Coverage of maternal health services varies, with approximately half covering prenatal care, 60 % basic delivery care, and 26 % complicated deliveries, including C-sections. [44].

Current evidence about CBHI illustrates modest achievements and enrolment challenges [46, 47], difficulty in reaching the poorest of the population [48], and challenges in financial and organisational sustainability [49]. However, the CBHI scheme can significantly contribute to financial protection, particularly if it is established as a complementary mechanism linked with social funds or the national health financing policy [46]. CBHI has been shown to increase both the demand for maternal health services and the rate of delivery with skilled birth attendants [50].

### Enhancing patient transfer

The common strategies to overcome difficulties of physically accessing available services are: emergency transport fund; provision of immediate alternative forms of transportation, and; provision of maternity waiting homes.

#### Emergency transport fund

Many communities have set up and administered loan funds for emergency obstetric transport to overcome difficulties in paying for transportation. This loan funds aim to tackle the problem of insufficient funds for healthcare by the poor. It is a local system established by communities for pooling and borrowing money to cover the transportation cost during emergencies [51].

Two studies conducted in Nigeria [52, 53] showed how communities could establish and manage emergency transport funds for maternal emergencies to reduce delay in accessing emergency obstetric care. Similar community-managed emergency transport funds were implemented in Pakistan [54], Bangladesh [55–57], and India [58]. Despite challenges, there is evidence that community transport funds and contracted transporters play a leading role in mobilising pregnant women to attend antenatal care and increase institutional delivery [43]. However, this depends on community leadership and considerable mobilisation efforts [59].

#### Intermediate forms of transportation

Facilitating geographical accessibility is crucial for access and utilisation of maternal care. There has been advocacy since the 1970s for appropriate intermediate modes of transport to health services in developing countries [60] that offer a locally appropriate and low cost mobility service in rural areas. Since then, many innovative, intermediate and alternative transport initiatives have been introduced to reduce the delay in referring women with maternal complications to health facilities, to reduce the cost and time of travel, and to link up with the referral system. These initiatives include motorised transport (such as motorcycles, pick-up trucks tractors and motorboats) and non-motorised transport (such as bicycles, animal drawn carts and canoes) [61]. For example, in Malawi three remote rural health centres were equipped with motorcycle ambulances to refer obstetric emergencies to the district hospital. Findings of this study found that motorcycle ambulances reduced referral delay by 35 %–76 %. Purchase price and operating costs were 19 to 24 times cheaper than for a car ambulance [62]. Linking the ambulance transportation with radio or telecommunications systems has improved referrals in many settings such as Burkina Faso [63] and Indonesia [64]. In rural Niger, prior to a radio–ambulance system, a woman with obstructed labour had no option other than to walk 75 kilometres or go by camel to reach the nearest hospital [65]. Non-motorised transport is slow, uncomfortable, occasionally culturally unacceptable and unfeasible for long distances [61]. For instance, deep cultural beliefs in rural Malawi deterred pregnant women from using a bicycle ambulance, designed to pull a wheeled trailer-stretcher, and reduced their utilisation of health facilities [66]. Recent reviews of transportation for maternal referral illustrate that motorised transports that consider cultural concerns are more likely to be an acceptable and effective choice for pregnant women during emergencies [61, 67].

#### Maternity waiting homes

Maternity waiting homes are residential facilities within easy reach of emergency obstetric care (EmOC) that aim to enhance access to care by bridging the geographical gap between women and services, and to increase institutional deliveries. These homes provide a place to stay and await labour for high-risk pregnant women or those who are living far away during the final weeks of their pregnancy. Those women have the opportunity to receive antenatal care and health education about pregnancy, delivery and neonatal care [68]. Some of these waiting homes are actually located within hospitals, as is the case in Ethiopia [69], or just next to the maternity ward, as in rural Zambia [70] and rural Timor-Leste [71]. Some of them are in accessible locations with secured transportation and communication facilities, such as some of the waiting homes in Cuba [72]. However despite studies that have reported positive effects of maternity waiting homes, utilisation of these facilities remains a challenge. Factors affecting satisfaction and utilisation include: quality of the facilities (small, crowded, poor hygiene) [73, 74]; cost of living (shortages of food, water and firewood, cost of reaching the hospital) [74, 75]; cultural issues, such as lack of awareness about the existence of the waiting homes, lack of privacy, inability to use traditional birthing practices, being away from the family and lack of respect from health staff [75, 76]; and access to services, issues here including safety concerns at night, cost of reaching the hospital and absence of healthcare personnel [75, 77]. A recent Cochrane review [68] that assessed the effects of a maternity waiting facility did not find any randomised controlled trials that evaluated the outcomes of maternity waiting homes in developing countries. The authors found wide variations between maternity waiting homes in term of facilities, location, population covered, capacity and level of care. In general there was limited reliability in terms of the study data that showed some favourable effects on outcomes for women and their newborns, but other studies indicating barriers to the utilisation of the facilities. There was insufficient evidence to determine the effectiveness of maternity waiting facilities.

### Community involvement

There are a number of approaches to overcoming demand-side barriers to accessing healthcare for improving maternal and reproductive health. These include specific community-based interventions, including community members, specifically women, in participatory learning and action on maternal health and insurance-based schemes.

#### Community-based interventions

Bringing healthcare to communities, through community participation and community-based interventions, is crucial for universal access to healthcare and for improving maternal and neonatal health [78]. Many approaches have been described including, for example, home visits, home management and facilitating referral [79]. Home visits involve promotion of birth and newborn-care preparedness via home-based antenatal care by female community health workers, and home-based postnatal care [80]. Another approach involves female or ‘lady’ health workers, who organise group sessions at the community to promote antenatal care, use of clean kits at delivery, institutional delivery, newborn care, danger signs identification and promotion of health-seeking behaviour [81]. In Pakistan for example, lady health workers (LHWs) from the communities in Hala and Matiari subdistricts are trained for 15 months to be able to identify all pregnant women in their area and provide to them basic antenatal care and maternal health education. They also promote use of clean delivery kits, encourage facility births and immediate newborn care. LHWs work in collaboration with voluntary community health committees and traditional birth attendants. LHWs are reimbursed for travel costs, but do not receive any financial motivation [81].

A recent Cochrane review [82] that included 18 cluster trials investigated the effects of community-based interventions in reducing maternal and neonatal morbidity and mortality and improving neonatal outcomes. The authors concluded that although skilled delivery and facility-based services for maternal and newborn care are important, the evidence is sufficient to recommend scaling up the community-based care packages.

Byrne *et al*. [83] in their systematic review of strategies to increase reproductive, maternal and child health in difficult to access mountainous locations categorised their findings into four main types of strategies. These included: training and improving serviced delivered through community health workers (CHWs); improving facility quality of care to make access more desirable; engaging communities; and improving health knowledge for timely care-seeking. In terms of engaging communities community-led planning was conducted in Bolivia through use of women’s groups in problem analysis, strategies and implementation of programmes resulting in improvements in health seeking behaviours. In Papua New Guinea health, education and agricultural representatives along with community leaders were involved in planning activities, though the impact of this was not evident .

#### Women’s groups practising participatory learning and action

Another promising approach involves women’s groups practising participatory learning and action. This includes a cycle of four phases: identification and prioritisation of maternal problems, planning for locally feasible solutions, implementation and assessment. A local woman facilitates each of these women’s groups and supports the women through a series of meetings. Interactive methods are used at these meetings, including stories, games and pictures, to discuss prevention, care seeking and treatment for common maternal and infant problems [84–86]. In Nepal for example, a female facilitator supports nine women’s group meetings each month in a population of 7000. She facilitates the group meeting through an action-learning cycle in which they identified local perinatal problems and formulated strategies to address them. These meetings enhanced demand for antenatal care and delivery by trained birth attendance. Birth outcomes improved greatly through this participatory intervention with women’s groups [84]. This bottom-up approach not only addresses how to reduce neonatal and maternal deaths but also addresses poverty, inequity, women’s empowerment and other social determinants for health [87]. A recently published systematic review and meta-analysis of the effect of women's groups practising participatory learning and action on improving maternal and newborn health in low-resource settings [88] confirmed that this approach substantially reduced neonatal and maternal deaths in rural and low-resource settings. These contextualised community-based interventions also led to significant behavioural changes and sustainable capacity development [58]. This method provides health education, based on dialogue and local problem solving, which is more effective and empowering than the message giving approach [88]. However, there are still unanswered questions, such as ‘What are the mechanisms behind the intervention effects?’ and ‘How best to promote participation?’ [87].

### Challenges that limit the effectiveness of demand side intervention at the community level

As discussed above, there are many interventions to enhance access to maternal health care [89]. Most of them are not linked in a programmatic approach, neither incorporated into a coherent planning and implementation process. One of the main factors contributing to the failure of maternal health programmes is the mismatch between the actual needs of the people and the circumstances in which healthcare is provided [90]. Numerous single interventions exist; however, no single intervention is by itself sufficient to improve maternal health and decrease morbidity and mortality [14]. These multiple interventions and projects often do not communicate, bypassing the government, and using standardised, inflexible models. Politicians, policy makers, health authorities, providers and target populations do not generally communicate with each other before developing maternal health programmes. Likewise, programme designers do not normally take into consideration the socioeconomic, cultural, political and other sensitive factors within the community when designing or implementing their programmes [90].

However, the major essential issues to increase demand for maternal health services in resource-limited settings are whether women have access to these demand-side interventions and whether they and their families benefit from them. This means that these strategies need to be effective at the community level. From our review of the strategies the main challenge is the lack of knowledge of the community for whom the strategies or interventions are being designed resulting in a lack of understanding of three fundamental influences on the decision making environment for the woman making the healthcare seeking decision. These influences are: Gendered decision-making norms, multigenerational dialogue, and appropriate communication.

### Gendered decision-making norms

Gendered norms determine sociocultural identity construction and attribution of rights and reflect unequal power relations. These norms affect risk and vulnerability, health-seeking behaviour and health outcomes as well as health sector responses of men and women of different ages and social groups [91]. Gender inequality is a cross-cutting determinant of health that operates in conjunction with other forms of discrimination. Gender norms that allow superior value and power to men increase women’s risk of gender-based violence, which can contribute to poor maternal health [92]. Unequal power in the decision-making process within household restricts women’s autonomy, limit her power to negotiate with her partner, increase fertility rates, increase unwanted pregnancy, and negatively affect maternal health [93, 94].

Women may not have access to household resources for health care, as family priorities may focus on household breadwinners, which are more likely to be male in many settings [95]. Gender norms demanding that girls should remain shy and innocent about sexual matters may limit their access to information on sexuality, contraception, pregnancy and related services [91]. Health care personnel may stigmatize and disrespect single mothers and pregnant adolescents. Judgmental personnel may prevent adolescents from accessing contraception or sexual and reproductive information or services [94]. In some settings, infertility is more likely to lead to shame, social ostracism or divorce among women than among men [96].

Gendered norms are inadequately considered in the design and implementation of demand side interventions and strategies to improve access to maternal healthcare. Maternal health is rarely examined through a gender lens resulting in strategies and interventions that do not improve access as the environment in which the decisions are being made remain unchanged.

### Multigenerational dialogue

There are multigenerational gaps existing between mothers and their daughters in which mothers are inhibited by social norms and values to create the opportunities for transferring of knowledge and experiences to their daughters resulting in lack of sexual education and preparedness for motherhood. Reproductive health issues are sensitive and usually not spoken of openly in society. Older generation women would rather not share motherhood experiences with their daughters due to cultural sensitivity and the association of the ‘young’ with ‘purity’ in which young unmarried girls should only explore reproductive health issues after marriage [97, 98]. This creates wide gaps in the knowledge across multiple generations, as experiences are not transferred down the family line. Daughters usually enter marriages at a young age and lack the knowledge, emotional and mental preparedness of motherhood [99]. These later result in young girls embarking onto motherhood with little knowledge on major life threatening danger signs associated with pregnancy and delivery. This profoundly reduces the quality of their maternal care experience especially in the early stages of motherhood [100].

Generational gaps are a growing reality in our modern world of more nuclear and urbanised households. With an increasing disconnection of urban households from their traditional roots conflict between traditional and modern attitudes and social beliefs can arise. Traditional values and perceptions within a community will gradually change. To incorporate change and to achieve consensus on best practice in motherhood intergenerational dialogue is a necessity. Without this dialogue conflict and discordance in maternal health practices within communities will continue. Bridging these gaps will enhance sustainability of positive changes and transferring the accumulated experiences. Challenges are further propelled by the absence life-cycle approach and resultant verticalisation of service delivery across the continuum of care from childhood to motherhood [92, 93].

### Appropriate communication

The lack of communication between pregnant women and health care providers is a challenge [101]. Many communities suffer from lack of utilization of accessible antenatal care [89]. The vast majority of antenatal care can be delivered by frontline providers including midwives, nurses, and community health workers, provided they have the necessary support and training. Frontline healthcare providers are an entry point to consistent maternal care after gaining the trust of women in the community. Antenatal care period needs to be a period of seized rather than missed opportunities, where not only are stronger links created but, where health information and services can significantly enhance the health of women and their infants.

Most of antenatal care booklets provide one-way communication, used as a method for dissemination of information, not designed in consultation with local women, and assume high level of literacy among pregnant women. Unidirectional communication weakens links between frontline health providers and women and leads to suboptimal maternal and neonatal health outcomes.

The recent series on midwifery in The Lancet [102] supports a system-level shift from focusing on “identification and treatment of pathology” to “skilled care for all” that strengthens the capabilities of women and values respect, communication, community knowledge, and understanding. Examples of capacity development for participation and joint planning with communities are few [83], but without understanding the knowledge base, practices and communication systems in a given community the chances of a ‘one size fits all’ approach to demand side interventions improving access to maternal health remain slim.

## Summary

Most of the demand side interventions and strategies aim to increase appropriate care-seeking (including antenatal care and institutional delivery) and appropriate home prevention and care practices for mothers and newborns. A few include some form of community engagement to contextualise the intervention and to overcome specific challenges faced by that community. However, it is rare that communities’ capacity and capability to be actively engaged in the planning, design and implementation of the intervention is described or discussed. Without this traditional decision-making practices will continue to disadvantage women, intergenerational conflict on best motherhood practices will not be addressed and inappropriate communication tools will be designed. There is a need to support pregnant women while embarking onto the motherhood journey, to harmonise the divergences maternal health practices, and to link pregnant women with younger generations. Context-friendly tools can assist in linking pregnant women with their peers, daughters, and with the frontline health worker and assist in strengthening community systems for improved maternal health. New mechanisms are also needed at community level to develop a more supportive maternal health environment that links with formal health system.
